# Animal Life in the Two Hemispheres

**Published:** 1834

**Authors:** 


### IV. Animal Life in the two Hemispheres.

We think that many of our readers will peruse with interest, the paper of our friend Dr. Lakey, on the causes of the great superiority, of the man of the northern, over the man of the southern hemisphere. Whatever may be thought of his theories, the facts which he has industriously collected and set forth, will be generally admitted; and have an obvious bearing on the natural and physiological history of our race.

